# Ethanol and anaerobic conditions reversibly inhibit commercial cellulase activity in thermophilic simultaneous saccharification and fermentation (tSSF)

**DOI:** 10.1186/1754-6834-5-43

**Published:** 2012-06-15

**Authors:** William R Kenealy, Christopher D Herring, David A Hogsett, Lee R Lynd

**Affiliations:** 1 Thayer School of Engineering, Dartmouth College, Hanover, NH, 03755, USA; 2 Mascoma Corporation, Lebanon, NH, 03766, USA; 3 Current address:Biosciences Center, National Renewable Energy Laboratory, 1617 Cole Blvd, Golden, CO, 80401, USA

**Keywords:** Cellulase inactivation, Anaerobic, Reduced environment, tSSF, High solids, GH61

## Abstract

**Background:**

A previously developed mathematical model of low solids thermophilic simultaneous saccharification and fermentation (tSSF) with Avicel was unable to predict performance at high solids using a commercial cellulase preparation (Spezyme CP) and the high ethanol yield *Thermoanaerobacterium saccharolyticum* strain ALK2. The observed hydrolysis proceeded more slowly than predicted at solids concentrations greater than 50 g/L Avicel. Factors responsible for this inaccuracy were investigated in this study.

**Results:**

Ethanol dramatically reduced cellulase activity in tSSF. At an Avicel concentration of 20 g/L, the addition of ethanol decreased conversion at 96 hours, from 75% in the absence of added ethanol down to 32% with the addition of 34 g/L initial ethanol. This decrease is much greater than expected based on hydrolysis inhibition results in the absence of a fermenting organism. The enhanced effects of ethanol were attributed to the reduced, anaerobic conditions of tSSF, which were shown to inhibit cellulase activity relative to hydrolysis under aerobic conditions. Cellulose hydrolysis in anaerobic conditions was roughly 30% slower than in the presence of air. However, this anaerobic inhibition was reversed by exposing the cellulase enzymes to air.

**Conclusion:**

This work demonstrates a previously unrecognized incompatibility of enzymes secreted by an aerobic fungus with the fermentation conditions of an anaerobic bacterium and suggests that enzymes better suited to industrially relevant fermentation conditions would be valuable. The effects observed may be due to inactivation or starvation of oxygen dependent GH61 activity, and manipulation or replacement of this activity may provide an opportunity to improve biomass to fuel process efficiency.

## Background

Processing cellulose to ethanol at high solids concentrations (e.g. >15%) is necessary for the economic viability of commercial processes [1], though most published studies of cellulase activity have been conducted at much lower concentrations. Operation at high solids concentration results in decreasing fractional conversion of the feedstock compared to operation at lower concentration. This ‘solids effect’ has been demonstrated in several cellulose hydrolysis processes, including enzymatic hydrolysis and simultaneous saccharification and fermentation (SSF) [1], and is not well understood.

Operation of SSF at higher initial solids concentrations is often accompanied by higher concentrations of soluble sugars and/or higher ethanol concentrations [1,2]. While these end products and solvents inhibit cellulase activity [3-5], this inhibition does not account for the total loss of activity seen under these high solid conditions [1]. Other factors that have been implicated in the observed slowdown in hydrolysis include enzyme inactivation [6-8], substrate inhibition [9,10], mass transfer [11,12], interference by lignin [13], loss of synergism and unproductive binding [14], inhibitors carried over from the feedstock [15] and changes in adsorption [1]. Yet as Kristensen and co-workers [1] demonstrated, none of these fully explained the declining activity across a spectrum of hydrolysis and SSF conditions, including a range of substrates and enzyme loadings.

Shaw et al. [16] reported the metabolic engineering of *Thermoanaerobacterium saccharolyticum -* a thermophilic, non spore-forming anaerobe that ferments cellobiose and hemicellulose but not cellulose – to produce ethanol at high yield. When SSF of 50 g/L Avicel was carried out with this organism and fungal cellulases at 50 °C, 2.5-fold less cellulase was required to get equivalent results compared to operation at 37 °C with the same enzyme using yeast as the fermenting organism. Subsequently, Podkaminer et al. [8] developed a kinetic model for tSSF with *T. saccharolyticum* ALK2, and found that the model worked well at describing experimental results at initial Avicel concentrations of 20 and 50 g/L.

When we carried out tSSF at an initial Avicel concentration of 77 g/L, we found that actual cellulose hydrolysis was less than that predicted by the model, indicating that there are phenomena operative at high solids concentration that we do not understand. The work reported here was undertaken with the objective of identifying such phenomena and explaining the larger-than-expected decrease in performance at high solids concentrations. In the process, we discovered a fundamental incompatibility of fungal cellulases with tSSF, which may have implications for the continued development of cellulase enzyme technology.

## Results

**Figure 1:**
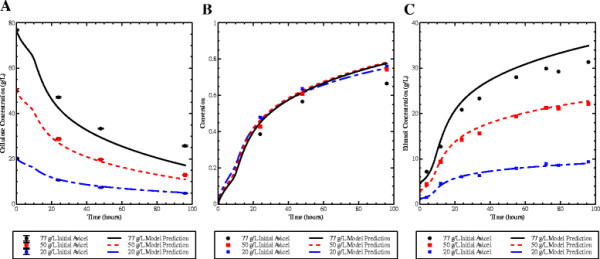
**tSSF performance at increasing initial solids.** Experimental data and model predictions from tSSF with 20 (blue, dashed), 50 (red, dotted), and 77 (black, solid) g/L initial Avicel, with 4 FPU/g cellulose. **A**) Cellulose. **B**) Conversion. **C**) Ethanol. Points indicate experimental data, lines show model predictions. Data indicate that the model predicts performance at 20 and 50 g/L initial Avicel, but over predicts experimental data at 77 g/L initial Avicel. Error bars in Panel A indicate the standard error of the cellulose concentration measurement.

Following the development of a model that matched tSSF performance at initial Avicel concentrations of 20 and 50 g/L [8], performance at 77 g/L was tested. As shown in Figure 1, the model matches well with experimental data at the lower initial Avicel concentrations, but not at 77 g/L.

Prior work in our group investigated the effect of ethanol on enzyme stability, which is subsequently incorporated into the described mathematical model of tSSF. However, even with this inactivation included, the model does not capture the decrease in conversion observed at higher initial solids concentrations in these experiments. In this combined hydrolysis and fermentation system, no accumulation of soluble sugars (glucose and cellobiose) was observed past 15 hours. Cellobiose remained below 0.2 g/L while the glucose concentration was below the level of detection (data not shown). This data indicate that enzymatic hydrolysis remains the rate-limiting step. Moreover, the observed concentration-dependent discrepancy was more pronounced at late time points rather than the initial stages of hydrolysis when soluble sugars were observed. Thus inhibition by hydrolysis products present in the bulk solution do not appear to be responsible for the lower-than-expected conversion at high solids.

**Figure 2:**
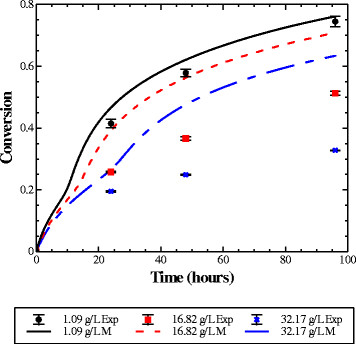
**Added ethanol tSSF.** Experimental data (points) and model results (lines) from 20 g/L initial Avicel concentration tSSF (4 FPU/g cellulose) supplemented with initial ethanol at concentration of 1.09 (black, solid), 16.82 (red, dashed) and 32.17 (blue dash-dot) g/L. The model over predicts conversion of 20 g/L Avicel in the presence of high concentrations of added ethanol. Error bars indicate standard deviation on the conversion measurement.

Higher initial cellulose concentrations lead to the production of higher ethanol concentrations. To isolate the effect of ethanol in tSSF without potential additional factors associated with higher solids concentration (e.g. higher cell mass, impeded mass transfer), tSSFs with initial Avicel concentrations of 20 g/L were supplemented with ethanol to initial concentrations of 1.07, 16.82, and 32.17 g/L (Figure 2). At a low initial ethanol concentration of 1.09 g/L, 75% of the initial Avicel was hydrolyzed. However, ethanol had a profound inhibitory effect on cellulase activity. With 32.17 g/L ethanol added at the beginning of fermentation, only 32% of the Avicel was converted to ethanol in 96 hours, yet the model predicts over 60% conversion. We previously measured both the inhibition and inactivation of cellulase activity due to ethanol in enzymatic assays at ethanol concentrations from 0 to 80 g/L, and these effects are accounted for in the tSSF model. Based on these measurements of initial rates, 50% of cellulase activity was expected at an ethanol concentration of 37.6 g/L. In addition, the model projects only a 15% difference in final conversion between tSSFs with an initial Avicel concentration of 20 g/L supplemented with initial ethanol concentrations ranging from 1.09 to 32.17 g/L. However, the measured data show over 50% loss in final conversion in tSSF due to the added ethanol and thus the negative effect of ethanol is greater than previously understood by enzyme assays.

A potential explanation for the discrepancy between the model prediction and empirical data is that in the development of the tSSF model, the effects of ethanol were characterized in buffer solutions exposed to air, rather than in the anoxic conditions of tSSF. The medium components as well as the highly reduced conditions of the *T. saccharolyticum* fermentation broth may contribute to this difference. Since the effect of added ethanol in tSSF was different from expectations based on previous assay conditions, the effects of the medium and the anaerobic environment were further investigated.

**Figure 3:**
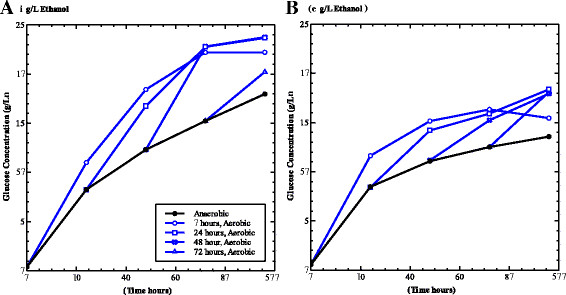
**Effect of exposure to air on hydrolysis in spent medium.** The effect of an anaerobic environment was tested by assessing glucose concentrations produced from the hydrolysis of 50 g/L Avicel in spent MTC medium in the presence of **A**) low ethanol (3 g/L) and **B**) high ethanol (40 g/L). The glucose production of samples maintained in anaerobic conditions for the course of the experiment (black, closed) was compared with samples transferred to aerobic conditions (blue, open) at 0 hours (circle), 24 hours (square), 48 hours (cross) and 72 hours (triangle). Results indicate exposure to air increased glucose concentrations at all sample time points. Data points represent individual samples but are representative of repeated experiments.

To systematically compare cellulose hydrolysis in an anaerobic versus an aerobic environment without adding chemical reductants, spent MTC medium was harvested from cultures in an anaerobic glove bag, and cellulose hydrolysis was then measured in this spent medium to mimic the conditions in tSSF. Immediately after cellulase addition and each subsequent 24 hours, a portion of this anaerobic reaction mixture was transferred to an air-filled vial to test the effect of headspace composition on cellulase activity. This test was conducted both at a low ethanol concentration (3 g/L, a result of ethanol in the spent medium as well as in the antibiotic solution) as well as at a high ethanol concentration of 41 g/L to see if the effects were synergistic. At both ethanol concentrations, cellulose hydrolysis proceeded more slowly under anaerobic conditions than aerobic conditions (Figure 3a, b). In order to rule out protease activity as the underlying cause, control experiments were performed comparing hydrolysis in spent medium to that in uninoculated, fresh anaerobic medium. Both conditions showed a pattern of similar glucose concentrations over time and an increase in hydrolysis upon aeration, ruling out the potential of protease activity in spent medium (data not shown). Thus, the clear difference observed between cellulose hydrolysis in aerobic and anaerobic conditions was due to the anaerobicity itself. All samples which were transferred from an anaerobic bottle to an aerobic bottle displayed a boost in glucose production regardless of the time at which those samples were transferred. In addition, glucose production of samples transferred at later time points approached glucose production of bottles that were transferred at time zero. Thus, this cessation of cellulase activity under anaerobic conditions is reversible. At both ethanol concentrations the aerobic conditions led to roughly 30 percent greater glucose production.

**Figure 4:**
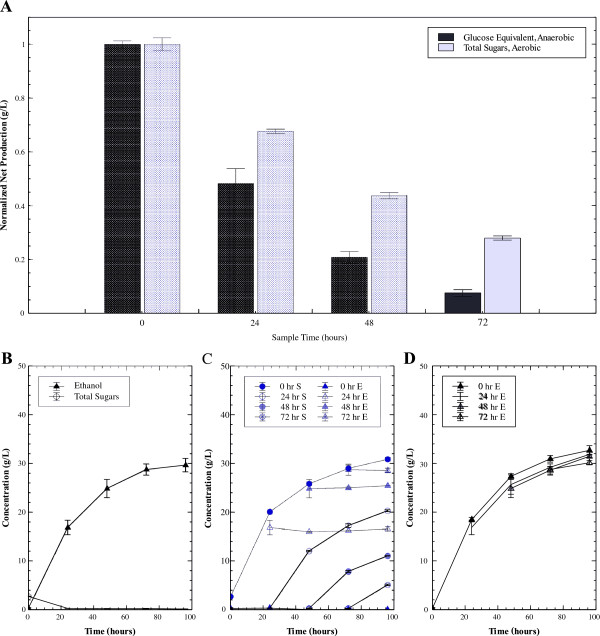
**Effect of aeration on tSSF.A**) 24 hour activity of samples drawn from 80 g/L Avicel tSSF (panel B) and transferred to either nitrogen or air-filled serum bottles at 0, 24, 48 and 72 hours, normalized to 0–24 hour production under respective conditions. **B**) Sugar and ethanol concentrations from an 80 g/L Avicel tSSF. **C**) Total sugar (cellobiose plus glucose) and ethanol concentrations measured in air-filled serum bottles after sampling from tSSF. Ethanol (E, triangles) and sugar (S, circles) from samples transferred at 0 (filled), 24 (open), 48 (shaded), and 72 (diagonal cross) hours. **D**) Ethanol concentrations produced in samples from tSSF transferred to nitrogen-filled serum bottles. Overall, the introduction of air to tSSF samples slowed the rate of cellulase inactivation compared to samples maintained under anaerobic conditions. Error bars indicate standard error between replicate bottles.

**Figure 5:**
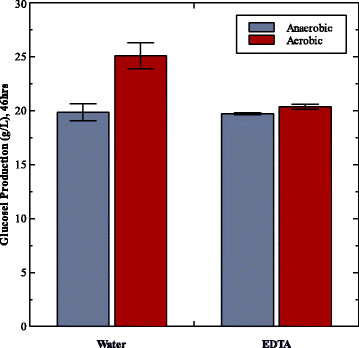
**Effect of exposure to air on hydrolysis in spent medium in the presence of EDTA.** Glucose produced (g/L) after 46 hours from the hydrolysis of 50 g/L Avicel in spent medium with 4 g/L ethanol under aerobic and anaerobic conditions, with and without 5 mM EDTA. Glucose production under anaerobic conditions shown in blue, under an aerobic headspace in red. Thus, the presence of EDTA prevented the increase in hydrolysis by exposure to air. Error bars indicate standard error of the mean for duplicates.

To confirm the effect of aeration directly in a tSSF system, samples were removed every 24 hours over the course of the tSSF (Figure 4b) and transferred to sterile air-filled bottles (Figure 4c). As a control, samples were also drawn and placed into bottles purged with nitrogen gas to remove all oxygen (Figure 4d). Upon transfer to the air-filled bottles sugars immediately accumulated, while ethanol production continued at a declining rate in nitrogen-filled bottles. A direct comparison of cellulase activity in these two headspaces is challenging because ethanol is the product under anaerobic conditions and sugars are the product under aerobic conditions, but can be approximated by assuming constant theoretical ethanol yield. Figure 4a shows the relative amount of product formed in the 24 hours following removal from the tSSF. These values are expressed in glucose equivalents and normalized to the activity of samples removed at time 0 for the respective headspace. While activity declines both in the presence and absence of air, relative activity declines faster in the anaerobic environment than in the air atmosphere. Of note are the samples taken after 48 hours. During this time period in tSSF, little additional ethanol is typically formed and no sugars accumulate, indicative of poor hydrolysis. By contrast, samples transferred to an aerobic environment after 48 hours accumulate over 10 additional g/L total sugars in 48 hours, and thus indicate that cellulase activity increases with exposure to air. These data demonstrate that while the enzymes are not active at the end of tSSF, they have not been irreversibly inactivated. The hydrolysis experiments described in Figure 3 were repeated with and without 5 mM EDTA to investigate whether this oxygen-dependent sugar production was a result of GH61 activity. GH61’s are divalent metal-containing glycohydrolases that have been shown to be inhibited in the presence of EDTA [17]. Among the cellulase enzymes produced by *T. reesei*, GH61 is the only enzyme highly inhibited by EDTA (Matt Sweeney, Novozymes, personal communication 2011). Therefore, assessing this system with the addition of EDTA is a good indicator for GH61 activity. When EDTA was added to the reaction mixture, there was no increase in hydrolysis upon aeration (Figure 5), with glucose production in an aerobic environment comparable to the levels measured under anaerobic conditions.

## Discussion

Thermophilic SSF (tSSF) allows for cellulose hydrolysis at a temperature optimal for cellulase enzymes without the interference of soluble sugars, which have a well-documented inhibitory effect [3-5]. However, despite the advantage of reduced enzyme loading [16], tSSF also shows a decrease in final conversion with increasing solids concentration. The trend has also been demonstrated in both SSF and hydrolysis systems, where it has been attributed to several causes, including product inhibition and enzyme inactivation. One of the potential causes of this heightened enzyme inactivation at the higher solids concentration is the concurrent presence of higher ethanol concentrations. Despite excess substrate remaining, hydrolysis stops at a lower conversion in the presence of higher ethanol concentrations. However, the predictions from the tSSF model [8] which take into account both inhibition and inactivation of cellulase activity by ethanol as measured by standard procedures [3-5,8] cannot predict this loss of activity. The 32% conversion measured after 96 hours from the tSSF with an initial ethanol concentration of 32.17 g/L shows that inactivation of cellulase enzymes are occurring faster and to a greater extent than has previously been measured and thus predicted by the model. Based on this discrepancy between experimental and model results from the added ethanol tSSF experiments our understanding embodied in the former model is incomplete.

The initial assessment of cellulase stability as a function of ethanol concentration was carried out by initial rate measurements in aerobic conditions. Since these correlations did not predict the extent of inactivation measured from tSSF directly, the conditions specific to tSSF were further evaluated. The effect of the reducing conditions was assessed by comparing hydrolysis in spent medium under a nitrogen headspace to an aerated control. Prior controls had shown no difference between anaerobic conditions setup with uninoculated, anaerobic medium versus spent medium, thus ruling proteases or other *T. saccharolyticum* enzymes as the source of this reduced activity. Subsequent reactions were performed in spent medium to best mimic tSSF conditions. Regardless of incubation time and exposure to anaerobic conditions, by transferring the reaction to a headspace filled with air faster hydrolysis was achieved, thus reactivating the enzymes. Given longer incubation periods, we predict that all aerobic samples would reach the same total glucose production, indicative that there is a given fraction of the substrate for which this activity is vital.

The inhibitory effect of a nitrogen environment was confirmed in the aerated tSSF experiment. In contrast to the spent medium used in the hydrolysis experiments, the aerated tSSF samples have actively growing cells and successively higher ethanol concentrations. The data further show that the inhibitory effect of the reduced environment and nitrogen headspace is a reversible phenomenon and that it limits further hydrolysis.

The commercial cellulase mixture used these experiments is derived from *T. reesei*, an aerobic fungus. Cellobiohydrolase I, the most abundant protein produced by *T. reesei*, contains 12 disulfide bonds [18,19]. We hypothesized that the reduced state of the medium achieved by fermentation with *T. saccharolyticum* leads to the reduction of these disulfide bonds. Once the disulfide bonds are reduced to sulfhydryls, the protein is less stable, thus escalating the effects of ethanol, a denaturant. A difference in hydrolysis between oxygen and nitrogen environments was demonstrated several decades ago by Eriksson and co-workers [20]. Using culture supernatants from several cellulolytic species, an increase in hydrolysis was measured in an aerobic environment. In the case of *Trichoderma viride,* they reported a 2-fold increase in hydrolysis under an oxygen containing environment. Rather than an effect on the disulfide bonds in the proteins hypothesized above, their work indicated the activity of an oxidizing enzyme. This enzyme was thought to use an oxidative mechanism to promote swelling of the crystalline cellulose by breaking hydrogen bonds. Since this early work, several oxidative enzymes including cellobiose quinine oxidoreductase, lactonase and cellobiose oxidase have been described. In addition, several wood degrading fungi utilize an oxidative enzyme, cellobiose dehydrogenase (CDH) [21,22]. However, while the presence of CDH activity has been reported for *T. reesei* by Dekker [23], it was later questioned by Henriksson and co-workers [21]. At present the presence of CDH activity in commercial *T. reesei* cellulase preparations remains to be definitively demonstrated.

Another redox-active class of enzymes, GH61s, has recently received much publicity and their mechanism and function are still under investigation. Preliminary results indicated that GH61 enzymes, like several of the enzymes described above may utilize an oxidative mechanism to cleave cellulose [24]. Novozymes reported that GH61 enzymes increase the enzyme efficiency on pretreated substrates, but do not do so on Avicel [17]. Further work determined that this discrepancy was due to the absence of redox-active co-factors which were present in the pretreated biomass. When the soluble fraction of dilute acid pretreated biomass was added to pure cellulosic substrates, an increase in cellulose degradation was observed [24]. Thus, the reactivation of enzyme activity upon exposure to air, and thus a higher redox state, led us to investigate the potential role of GH61 enzymes in our system. Since copper is also necessary for GH61 stimulation [17,25,26], the addition of EDTA is a suggestive, though not definitive, means to test for GH61 activity. As shown in Figure 5, in our system the addition of EDTA prevented the reactivation of cellulase activity upon aeration. Since EDTA chelates metal ions, this result likely indicates that GH61 enzymes or the cellulase components they interact with are likely being inactivated in the anaerobic, reducing conditions of tSSF, though further work is necessary to confirm this hypothesis. In addition, the previously demonstrated ethanol inhibition, which the model could not predict, may also be a result of GH61 dependent inactivation.

Overall, while the precise mechanism underlying the loss of cellulase activity under nitrogen conditions is not known, the increase in activity upon exposure to air suggests that a redox dependent change is occurring. Whether the enzyme itself is altered due to the low redox state and/or hydrolysis itself utilizes an oxidative mechanism and thus does not function in a reduced environment, is still unknown. The opportunity to achieve higher conversion makes this phenomenon important to pursue.

## Conclusion

Both ethanol and the anaerobic, reduced environment play a role in slowing down and stopping hydrolysis in tSSF. The presence of ethanol results in greater inactivation than has been previously described by analysis of cellulase stability in buffer solutions alone. This is, in part, due to the combined effects of ethanol and a nitrogen headspace on fungal enzymes that have evolved in an oxygen environment. The work presented here shows faster loss of activity at higher solids concentration as well as two factors contributing to enzyme inactivation, high ethanol concentration and a reducing environment. These data suggest the need for enzymes suited to the anaerobic fermentation conditions attained in ethanol production. In addition, data presented here indicate GH61 enzymes may be important for cellulose hydrolysis in tSSF, highlighting the potential challenges for tSSF processes. The conditions examined in this study provide a good model to explore the deficiencies in commercial cellulase mixtures as well as the specific effects of ethanol and a reduced, nitrogen environment.

## Methods

### Strains and cultivating conditions

*T. saccharolyticum* ALK2, constructed by Shaw et al. [16], was used for all experiments. The strain was maintained using stock cultures prepared from exponentially growing cells, which were stored with 5% v/v dimethyl sulfoxide (DMSO) at −80 °C.

### Medium formulation

The MTC medium was prepared as described in [27] with modifications as described in [8]. The carbon source and quantity are noted in the following sections. All medium components are from Sigma-Aldrich (St. Louis, MO) with the exception of the yeast extract (low dust yeast extract from BD Difco) used in the hydrolysis and aerated tSSF experiments and Avicel PH-105 (FMC, Philadelphia PA).

### tSSFs

Thermophilic SSFs were performed with *T. saccharolyticum* ALK2 as described previously, [8]. At the time of inoculation, cellulase enzymes (Spezyme CP, Genencor 159 FPU/ml) were added at 4 FPU/g cellulose. No beta-glucosidase was supplemented as *T. saccharolyticum* ALK2 utilizes cellobiose. Solids were suspended by agitation at 150 RPM. The pH of the fermentation, which was monitored throughout the fermentation, stayed at 5.0 ± 0.05 without active control. Samples used to assess residual cellulase activity were drawn from 20 and 80 g/L initial Avicel concentration tSSF. Upon sampling, a 1 ml sample was separated into supernatant and pellet fractions. The supernatant, pellet and total samples were frozen for subsequent residual activity measurements. Added ethanol tSSFs were run at 20 g/L initial Avicel concentration with exogenous ethanol added prior to inoculation. Total initial ethanol concentrations, corresponding to the sum of exogenous ethanol added plus ethanol carried over from the inoculum, were 1, 17 and 32 g/L. Conversion was determined by residual cellulose measurements using quantitative saccharification [28].

### Residual enzymatic activity

To determine the amount of enzyme activity retained over the course of tSSF, samples drawn from the 20 and 80 g/L initial Avicel tSSF were assayed aerobically for residual activity. Upon sampling, the supernatant and pellet were separated by centrifugation and frozen. Thawed supernatant and pellet samples were diluted back to the original concentration with 50 mM citric acid buffer. Samples were further diluted with 50 mM citric acid buffer to give comparable enzyme concentrations (w/v) between samples from the high and low Avicel concentration tSSFs, while also reducing background ethanol concentration. Samples from 20 g/L initial Avicel were diluted 5-fold, while 80 g/L samples were diluted 20-fold. Residual enzymatic activity of supernatant and pellet samples, as well as an independent total sample, was measured by the production of reducing sugars as described by Podkaminer et al. [8] with the following changes: Incubation time was increased to 8 hrs. DNS reagent was modified to include 2 g/L phenol. Reducing sugars were quantified by reacting the total assay sample (both solids and supernatant) with DNS reagent. The solids were then pelleted by centrifugation and absorbance of the DNS supernatant was measured at 540 nm. Percent residual activity was calculated relative to the initial activity of the total sample at the respective solids level.

### Hydrolysis experiments

Hydrolysis of 50 g/L Avicel was monitored in spent MTC medium. Spent medium was produced by growing *T. saccharolyticum* ALK2 on 5 g/L cellobiose MTC medium in an anaerobic glove bag (Coy Laboratory Products, Grass Lake, MI) in the presence of 62.5 g/L Avicel. After the utilization of the cellobiose, the resultant spent medium was used as 80% v/v of the hydrolysis reaction resulting in an initial concentration of 50 g/L Avicel. Unspent medium was prepared as described above but without a carbon source or inoculum. A mixture of antibiotics was added to the hydrolysis reaction to prevent microbial growth with final concentrations of: penicillin 60 μg/ml, streptomycin 50 μg/ml, ampicillin 50 μg/ml, kanamycin 200 μg/ml, chloramphenicol 200 μg/ml, erythromycin 10 μg/ml, tetracycline 10ug/ml. Sodium acetate (50 mM, pH 5.0) was used to buffer the medium. Total ethanol concentrations (ethanol from spent fermentation medium, plus added ethanol and ethanol from antibiotic solutions) were 4 and 41 g/L. Cellulase enzymes (Spezyme CP) were added at 4 FPU/g cellulose and supplemented with 40 IU/g cellulose of beta-glucosidase (Novozyme 188, Sigma). Hydrolysis reactions were prepared in serum bottles at 40 mls in an anaerobic chamber and sealed with butyl stoppers. One half of the reaction mixture (20 mls) was immediately removed and transferred into air-filled serum bottles and sealed. Both samples were then incubated at 50 °C and shaken at 150 RPM for 4 days. Samples were drawn every 24 hours and analyzed by HPLC for sugar and ethanol concentrations (Bio-Rad Aminex 87-H).

To assess for the presence of GH61 activity, the hydrolysis of 50 g/L Avicel in spent medium described above were repeated with and without 5 mM EDTA at the low ethanol (4 g/L) level. Samples were withdrawn at 0 and 46 hours and analyzed for glucose production via HPLC.

### Aerated tSSFs

Aerated tSSFs were run as described above with an initial Avicel concentration of 80 g/L, except the fermentation was seeded with a 5% v/v inocula of *T. saccharolyticum* ALK2 grown overnight on MTC medium with 20 g/L cellobiose and 30 g/L maltodextrin. To compare the effect of an aerobic versus anaerobic environment, at time zero and every subsequent 24 hours, 20 ml samples were taken in duplicate and transferred to sterile 125 ml serum bottles sealed with a butyl stopper containing either air or N_2_. Bottles were then placed in a 50 °C incubator, shaken at 150 RPM, for continued incubation and sampled every 24 hours. Products were analyzed via HPLC.

### Mathematical modeling

The mathematical model of tSSF, with parameters fit to data at low solids concentrations and described in Podkaminer et al. [8], was used to predict performance of tSSF at high solids concentrations and with added ethanol. The model incorporates rate equations for cellulose, cellobiose, glucose, ethanol and cell concentrations. In addition, the inhibitory effects of sugars and ethanol as well as an ethanol-dependent inactivation of cellulase activity are built into the model. Computer modeling was performed using Berkeley Madonna, a differential equation solving software.

## Abbreviations

SSF, Simultaneous saccharification and fermentation; tSSF, Thermophilic simultaneous saccharification and fermentation; GH61, Glycoside hydrolase family 61; FPU, Filter paper units; MTC, Medium for thermophilic clostridia; CDH, Cellobiose dehydrogenase.

## Competing interests

KP is a past employee and BK, DH, and CH are current employees of Mascoma Corp., for which LL is a Co-founder and Director. Mascoma has an active interest in technology for conversion of biomass to fuels.

## Authors’ contributions

KKP performed the work presented herein and drafted the manuscript. WRK, CDH and DAH supervised the work and assisted in experimental design. LRL conceived of the study, supervised the work and assisted in drafting the manuscript. All authors have approved of the final manuscript.
